# Multiplexed emitting system for an energy-recovery-linac-based coherent light source

**DOI:** 10.1107/S1600577523008263

**Published:** 2023-10-19

**Authors:** Lu Cao, Junhao Liu, Zhen Wang, Dazhang Huang, Chao Feng, Zhentang Zhao

**Affiliations:** a Shanghai Institute of Applied Physics, Chinese Academy of Sciences, Shanghai 201800, People’s Republic of China; b University of Chinese Academy of Sciences, Beijing 100049, People’s Republic of China; c ShanghaiTech University, Shanghai 201210, People’s Republic of China; d Shanghai Advanced Research Institute, Chinese Academy of Sciences, Shanghai 201210, People’s Republic of China; RIKEN SPring-8 Center, Japan

**Keywords:** energy recover linac, multi-beamline operation, coherent radiation, angular-dispersion-induced microbunching, multi-bend achromat

## Abstract

Recently, a novel approach has been proposed to produce ultrashort, fully coherent high-repetition-rate EUV and X-ray radiation by combining an energy recovery linac (ERL) with the angular-dispersion-induced microbunching method­ology. It is critical to maintain microbunching when the beam passes through bending magnets between the undulators, which results in difficulties supporting multiple beamlines. In this paper, the design of a multiplexed emitting system consisting of multi-bend achromats, matching sections and radiators to facilitate the multi-beamline operation is presented. Theoretical analysis and numerical simulations have been carried out and the results show that the microbunching and beam quality can be well maintained after four times of bending. Five radiation pulses with a central wavelength of 13.5 nm and peak power at the MW level have been produced by the same electron beam via this multiplexed emitting system. The proposed method holds potential in the multi-beamline operation of ERL- or storage-ring-based coherent light sources.

## Abstract 2

The design of a multiplexed emitting system based on an energy recovery linac is presented. Five radiation pulses with a central wavelength of 13.5 nm and peak power at the MW level have been produced by the same electron beam via this system.

## 1. Introduction

In principle, a light source based on an energy recovery linac (ERL) has virtues of both storage ring and free-electron laser (FEL), providing short duration, high spatial coherence and high-average-power radiation pulses (Bartnik *et al.*, 2020 ▸; Ben-Zvi, 2016 ▸; Gruner *et al.*, 2002 ▸). An ERL is capable of recovering electron beam power with nearly perfect efficiency in the main linac. The recycled power will accelerate subsequent electron bunches, ensuring a fresh electron beam in each turn (Arnold *et al.*, 2020 ▸; Litvinenko *et al.*, 2006 ▸). The initial idea of the ERL originates from Tigner (1965 ▸) and was firstly demonstrated in a superconducting radio-frequency (SRF) linac at Stanford University (Smith *et al.*, 1987 ▸). In ERLs, the electron beams experience a relatively short time in the accelerator compared with that in the storage rings, avoiding many effects that lead to emittance growth or depolarization, as well as equilibrium with emittance dilution mechanisms (Ben-Zvi, 2016 ▸; Gruner *et al.*, 2002 ▸; Smith, 2006 ▸). By providing low energy spread, low emittance and ultra-short electron beams, ERLs have the potential capability of expanding the field of energy sciences to new depths.

A number of SRF ERL facilities have been constructed or commissioned worldwide, such as the THz/IR/UV photon source in JLAB (Neil *et al.*, 2000 ▸, 2006 ▸), the compact ERL in KEK (Akemoto *et al.*, 2018 ▸), the CBETA facility in Cornell and BNL (Hoffstaetter *et al.*, 2017 ▸) *etc*. However, it is worth pointing out that the average current (tens of milliamps) achievable in ERLs is approximately one order of magnitude lower than that in storage rings (hundreds of milliamps) (Shen *et al.*, 2021 ▸), primarily as a consequence of the beam breakup effect in the SRF linacs (HOM effects) (Tennant *et al.*, 2005 ▸; Hoffstaetter & Bazarov, 2004 ▸) and the photoinjector (Dunham *et al.*, 2013 ▸). Besides, due to the difficulty of maintaining electron beam quality in the arc section, the peak current of the electron beam in the ERL is still not high enough to initiate a high-gain FEL at short wavelength (Kondratenko & Saldin, 1980 ▸; Bonifacio *et al.*, 1984 ▸).

Recently, a novel electron beam manipulation technique termed angular-dispersion-induced microbunching (ADM) (Li *et al.*, 2020 ▸; Feng & Zhao, 2018 ▸) has been proposed, which utilizes small laser-induced energy modulation to directly imprint strong coherent microbunching onto electron beams, significantly reducing the requirement on laser power. Consequently, a new complex (Zhao *et al.*, 2021 ▸) has been proposed to generate fully coherent radiation pulses with high repetition rates by implementing the ADM technique on the ERL. The combination of ADM and ERL can take full advantage of the GHz-level repetition rate, full coherence and high-flux with a sub-meV spectral resolution compared with the storage ring (Zhao, 2010 ▸) and the FEL (Madey, 1971 ▸; Emma *et al.*, 2010 ▸). Three-dimensional (3D) start-to-end (S2E) simulations have been carried out with feasible parameters of the ERL and the results show that fully coherent radiation pulses can be achieved with an average brightness approximately five to six orders of magnitude higher than that of the diffraction-limited storage rings (Zhao *et al.*, 2021 ▸; Eriksson *et al.*, 2014 ▸; Einfeld *et al.*, 2014 ▸), making the proposed scheme a potential candidate for the next generation of light sources.

To support multi-beamline operations and improve the efficiency of the light source, it is necessary to incorporate electron beam bending systems between the undulators. Currently, multi-beamline operation studies primarily focus on storage rings (Cai *et al.*, 2012 ▸; Labat *et al.*, 2018 ▸) and FELs (Rönsch-Schulenburg *et al.*, 2017 ▸; Fröhlich *et al.*, 2019 ▸). Those based on ERLs are realized through multi-turns (Neil *et al.*, 2006 ▸; Shevchenko *et al.*, 2016 ▸; Angal-Kalinin *et al.*, 2018 ▸), which pose challenges in terms of occupancy and cost. Moreover, most multi-emitting operations do not consider the re-use of the same beam and microbunching preservation. In 2010, Li *et al.* (2010 ▸) proposed several bending system solutions for the European XFEL, which can preserve the microbunching throughout the bend. However, the bending angles in that study were on the 100 µrad scale, which may be insufficient for compact structures to separate radiations and bend the beam, and only a single deflection was demonstrated.

In this paper, a feasible multiplexing emitting system for the ERL-based coherent light source is proposed. Multi-deflection of the same beam while preserving microbunching throughout the entire operation based on the ERL has been realized with the proposed scheme. The total bending angle can be increased to 8 mrad, allowing for at least four deflections to support five high-intensity EUV pulses which significantly improves the radiation efficiency. S2E 3D numerical simulations have also been performed to evaluate the EUV radiation performance and demonstrate the feasibility of the proposed scheme. The rest of this paper is organized as follows: the physical design is given in Section 2; in Section 3 the S2E 3D simulations are carried out to show the performance of the proposed scheme; and a summary is given in Section 4.

## 2. Design for the multiplexing emitting system

**Figure 1:**
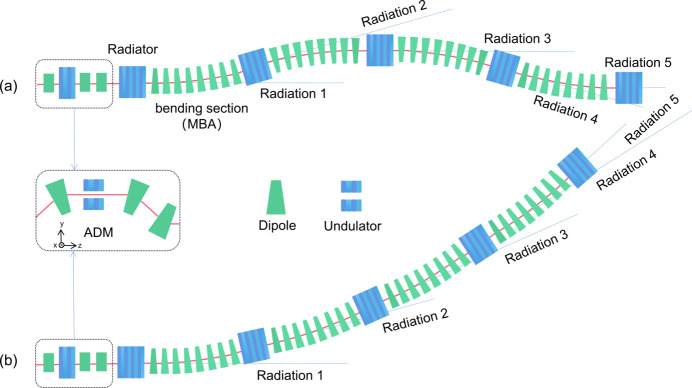
Two possible layouts for the multiplexing system.

We propose two possible multiplexing geometries that both incorporate an ADM section, bending systems and radiators, as shown in Fig. 1 ▸. The energy modulation and density modulation are generated when the electron beam passes through the ADM section. Subsequently, fully coherent, short-duration and high-power radiation pulses are generated in the downstream bending-radiator systems to support multi-beamline operation. The electron beam is deflected vertically in the ADM section and horizontally in the bending system. Considering practical operations, such as the consistent commissioning of magnet strengths, implementing identical bending lattices at each bending position emerges as a more feasible option. In the first scenario [Fig. 1 ▸(*a*)], the upstream and downstream trajectories of the electron beam are parallel, and radiators 1, 3 and 5 are in parallel with a separation of 16 cm in the horizontal. This scheme can be inserted in the drift section of the ERL. In the second scenario [Fig. 1 ▸(*b*)], the electron beam is kicked in the same direction with a total bending angle of 32 mrad. This scheme can be inserted into the arc of the ERL.

The purpose of designing the bending system is to transport and deflect the pre-bunched electron beam several times without distorting its longitudinal and transverse phase space distribution. Here, a lattice design is adopted based on the FODO cell, which is capable of forming a second-order achromat without the assistance of sextupoles (Sun, 2011 ▸). Moreover, all other second-order terms must be eliminated to mitigate the distortion in longitudinal phase space distribution. The transverse transport matrix elements are classified as geometric terms (depending upon the central momentum) and chromatic terms (depending on the momentum deviation). The second-order geometric aberration terms can be expressed as (Brown & Servranckx, 1985 ▸) 

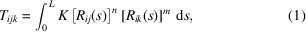

where (*n* + *m*) = 3, and *K* refers to the strength of the magnet elements at different locations. Here, quadrupoles do not contribute to the geometric aberration. *R*_*ij*_(*s*) are the first-order terms and are a linear combination of sinΔφ and 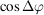 , and equation (1) can be written as 

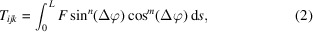

where the functions *F* are equal to the strength *K* multiplied by some power by the β(*s*) functions. Thus, to eliminate all second-order geometric terms, the following conditions must be met,

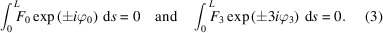

The integral of the expression Fe^±*i*φ^ and Fe^±3*i*φ^ for each separate element of a lattice can be represented geometrically as a vector in the complex plane. Thus, equation (3) can be written as 

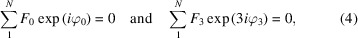

where *N* is the cell’s number. For repetitive FODO cells, *F*_*k*_ functions are equal to each other at the same location from cell to cell. With at least four repetitive cells and a total phase advance of 2π, the vector sum of the geometric terms will be zero.

In conclusion, in a lattice consisting of several periodic identical cells with the phase advance of 2π, all second-order geometric terms introduced by the dipoles and sextupoles can be compensated exactly, which can be realized by adjusting the quadrupoles’ strength and the drifts’ length (Brown & Servranckx, 1985 ▸). Additionally, sextupole families should be inserted into the FODO cell to eliminate the chromatic terms. One can tune the strength of sextupoles to eliminate one of the second-order chromatic terms (such as *T*_1*j*6_ and *T*_3*j*6_), indicating that all the second-order chromatic terms will become zero (Carey, 1981 ▸). Up to now, only *T*_5*ij*_ do not vanish, 

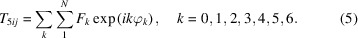

For repetitive cells, equation (5) can be written as 

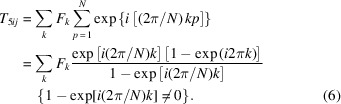

Since *k* is an integer, *T*_5*ij*_ will be equal to zero in the case of 1 − exp[*i*(2π/*N*)*k*] ≠ 0. Therefore, *N* must be larger than *k*. So, to eliminate *T*_5*ij*_ except *T*_566_, at least seven identical cells are needed (Li *et al.*, 2010 ▸).

**Figure 2:**
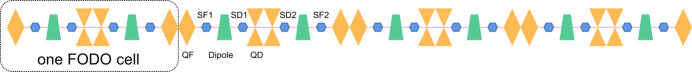
Layout of multi-bend achromat structure.

According to the theoretical analysis described above, the bending system can be designed as an achromat consisting of four FODO cells. This layout of this configuration is depicted in Fig. 2 ▸. Because of symmetry, each FODO cell can be seen as two identical cells. To remove all second-order chromatic terms, each FODO cell includes two sextupole families. The phase advances between each family can be optimized to make all the vector sums of new second-order geometric aberrations introduced by sextupoles cancel out (Brown & Servranckx, 1987 ▸). In 2011, Y. Cai developed an innovative method to calculate the resonance driving terms of an arbitrary order along a beamline. This method can be applied to study the resonance terms driven by sextupoles (or multipole families of sextupoles) and the results reveal that cancelations of high-order resonances are extremely general in an achromat (Cai *et al.*, 2012 ▸). In an achromat consisting of eight cells, the cancelations only rely on the cell’s phase advances and its periodic property. The cell phase advances can be chosen as μ_*x*_ = 3π/4 and μ_*y*_ = π/4 (Cai, 2011 ▸). In our case, the cell (half of the FODO) phase advances are chosen as μ_*x*_ = π/4 and μ_*y*_ = π/4.

In theory, all the first- and second-order terms can be canceled out except *R*_56_ and *T*_566_ after meeting the above demands. Besides, to ensure feasibility of the proposed scheme and obtain a starting point for smaller *R*_56_, it is also necessary to calculate the first-order matrix of the entire multi-bend achromat (MBA) before full simulation. The simplified first-order transfer matrices of the dipoles, the focusing quadrupoles, defocusing quadrupoles and the drifts are

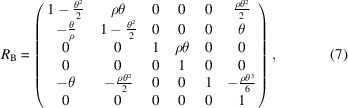




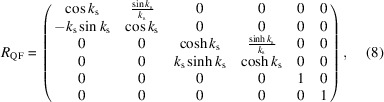




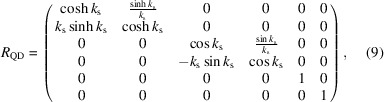




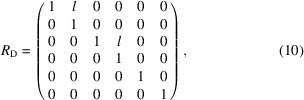

where θ and ρ are the bending angle and the radius of the dipole, respectively, *k*_s_ is the square root of the focusing quadrupole’s strength and *l* is the length between the centers of the adjacent quadrupole and dipole. Here, θ is set as 1 mrad and the length of the dipole is 0.15 m.

**Figure 3:**
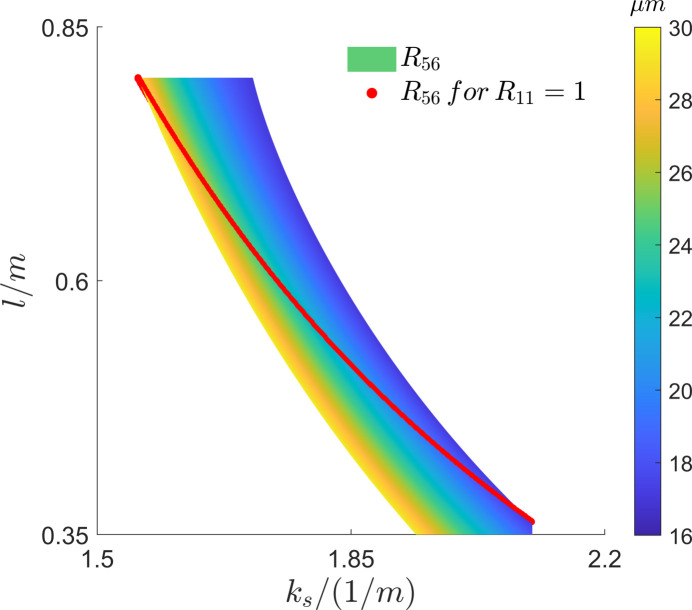
*R*_56_ values at different *k*_s_ (the square root of the focusing quadrupole’s strength) and *l* (the length between the centers of the bends and the quadrupoles). Here, the red dots represent *R*_56_ values calculated using the corresponding *k*_s_ and *l* when *R*_11_ is equal to 1.

Thus, the transfer matrix of the FODO cell can be calculated as *M*_FODO_ = *R*_QF_·*R*_D_·*R*_B_·*R*_D_·*R*_QD_·*R*_QD_·*R*_D_·*R*_B_·*R*_D_·*R*_QF_ and that of the total bending system is *M* = *M*_FODO_·*M*_FODO_·*M*_FODO_·*M*_FODO_. *R*_56_ of the bending system can be expressed using *k*_s_ and *l*. As shown in Fig. 3 ▸, the corresponding *R*_56_ values were calculated for a phase shift of 2π, *i.e.* *R*_11_ = 1. The trend change in *R*_56_ can be seen clearly in the figure, where small *l* values with large *k*_s_ values correspond to lower *R*_56_ values. The lowest *R*_56_ value is about 17 µm and the highest one is about 29 µm. Considering that the sextupole’s length is set to be 0.25 m and the gap between magnets needs to be reserved, the smallest value possible for *l* is set to be 0.45 m, and the corresponding square root of the focusing quadrupole’s strength is about 1.945 m^−1^.

In this case, the *R*_56_ value is approximately 19.5 µm. It is worth mentioning that higher bending angles call for higher quadrupole strengths to obtain proper *R*_56_. Similarly, higher sextupole strengths are also needed to eliminate second-order aberrations. In view of the balance among the magnets, a bending angle of 1 mrad is chosen. Moreover, this angle is sufficient to separate the radiation and bend the beam on a millimetre scale, making it suitable for our case and easily observable in a practical machine.

Following the theoretical calculation, the initial parameters of the dipoles, drifts and quadrupoles have been determined. These values will be used as the starting point in *ELEGANT* (Borland, 2000 ▸) to optimize for phase advances and Twiss parameters with a relatively short period of time.

Overall, this procedure enables quick determination of starting values and feasibility estimation. In addition, subsequent optimization is relatively straightforward due to the limited number of optimal objects and objectives for the symmetric lattice. These aspects make the lattice design flexible for radiations with different wavelengths.

## 3. 3D simulations

To demonstrate the performance of the bending system, S2E 3D numerical simulations were conducted with the electron beam parameters (Zhao *et al.*, 2021 ▸) presented in Table 1 ▸. The beam, with normalized emittance of 0.5 mm mrad, energy of 15 MeV, peak current of 15 A and pulse charge of 77 pC, is achieved by the end of the injector. The initial slice energy spread in the injector ranges from 1 to 3 keV (Feng *et al.*, 2011 ▸). The round-to-flat technique is utilized at the injector exit, with the normalized horizontal emittance being 100 times larger than the vertical emittance. This choice is based on the fact that the bunching factor of ADM depends on a lower RMS intrinsic vertical divergence, making a lower vertical emittance beneficial for high harmonic generation in ADM. Following the main linac, the electron beam energy is enhanced to 600 MeV. The electron beam is then compressed to 100 A in the arc section with a compression factor of about six (Zhao *et al.*, 2021 ▸). Consequently, the slice energy spread (RMS) at the ADM entrance is around 10 keV.

The subsequent optimization of the MBA cell was carried out with the tracking codes *ELEGANT* and *BMAD* (Sagan, 2006 ▸), attempting to obtain the desired phase advances and Twiss parameters, eliminating both the geometric and chromatic terms. In the virtues of symmetry, only six elements need to be tuned, significantly reducing the optimization difficulty. Only the dipoles and quadrupoles are considered in the incipient optimization. The strength of the quadrupoles (QF and QD) and the length of the drifts are fine-tuned on the basis of theoretical values to achieve a total 2π phase advance and cancel out the geometric terms. In this case, several equations of these second-order terms can be obtained,

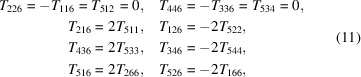

from which one can see that *T*_5*ij*_ is associated with the chromatic terms. *T*_126_ = 0 and *T*_346_ = 0 can be achieved by optimizing the strength of the sextupoles. Thus, all second-order terms are optimized to zero except *T*_566_. The optimized lattice parameters of the modified MBA are presented in Table 2 ▸.

To illustrate a potential application with actual parameters and to demonstrate the performance of the proposed scheme, 3D simulations were conducted with the tracking code *GENESIS* (Reiche, 1999 ▸) based on the beam parameters listed in Table 1 ▸. Firstly, the electron beam passes through the ADM section to interact with the seed laser with a wavelength of 256.5 nm to obtain energy and density modulation. Then the electron beam is transported into the radiator to generate the 19th harmonic radiation with the central wavelength of 13.5 nm. After that, the electron beam passes through the designed bending system, resulting in an 8 mrad deflection while maintaining the microbunching. Subsequently, the electron beam will travel through the radiator again to produce the second radiation with a central wavelength of 13.5 nm, followed by the second 8 mrad bending. To generate five radiations, the bending–radiator simulation process will be repeated five times in total.

**Figure 4:**
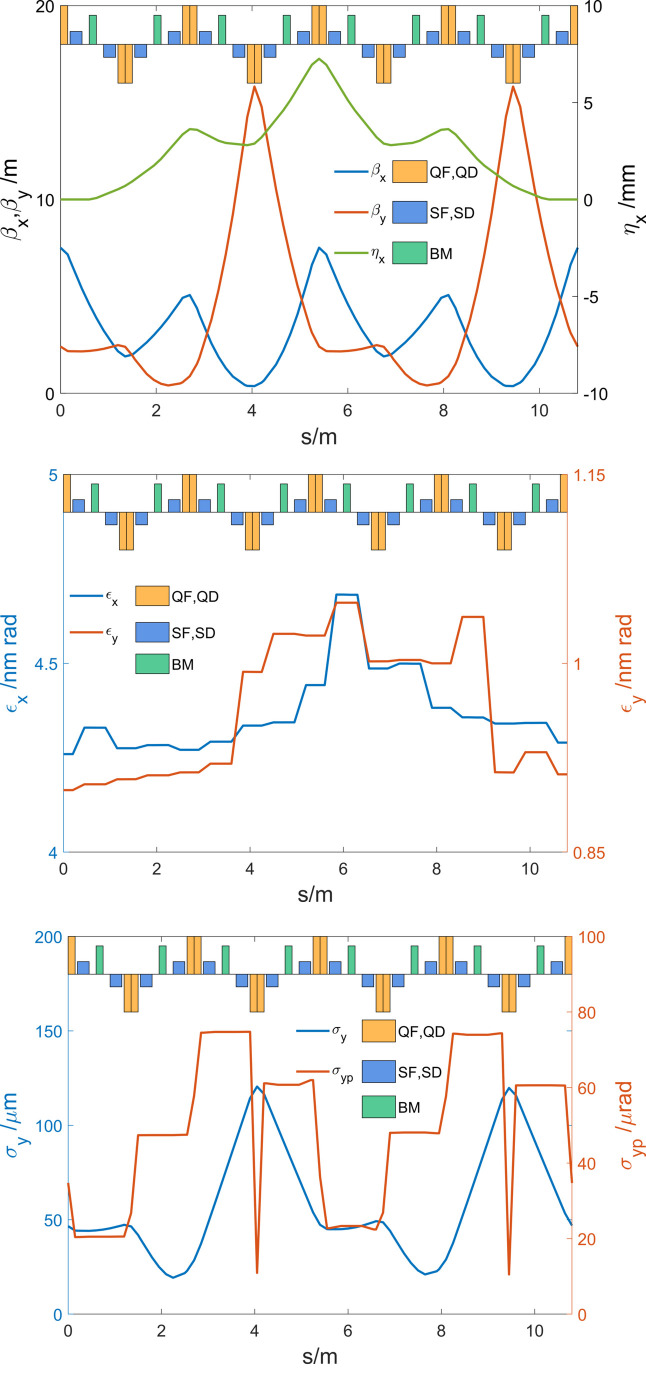
Twiss functions (top), emittance evolution (middle) and vertical beam size evolution (bottom) along the bending system.

*ELEGANT* is used to track the electron beam throughout the bending system with the second-order optical effects and coherent synchrotron radiation (CSR) effect taken into account. Figure 4 ▸ shows the Twiss functions (top), emittance evolution (middle) and vertical beam size evolution (bottom) along the bending system. The second-order aberrations from sextupoles play a significant role in exciting and canceling the emittances. Additionally, the CSR effect causes a 0.7% increase in horizontal emittance and a 0.14% rise in vertical emittance. As shown in Fig. 4 ▸ (bottom), the vertical dispersion inherited from the upstream ADM is effectively controlled due to the the 2π phase advance.

Considering the aforementioned settings, the quantitative RMS energy spread induced by CSR can be calculated using the following formula (Hajima, 2004 ▸; Bassi *et al.*, 2006 ▸),

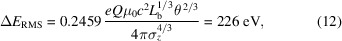

where *Q* is the bunch charge, μ_0_ is the permeability of vacuum, *c* is the speed of light, *L*_b_ is the bending path length, θ is the bending angle and σ_*z*_ is the RMS bunch length. It is worth noting that the induced RMS energy spread is two orders of magnitude smaller than that of the electron beam in the bending system. The electron beam position offset caused by CSR effect can be calculated as 

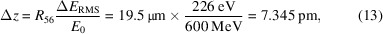

where *E*_0_ is the electron beam energy. The calculation value amounts to approximately 0.05% of the radiation wavelength, a minuscule magnitude that exerts negligible influence on the bunching performance.

**Figure 5:**
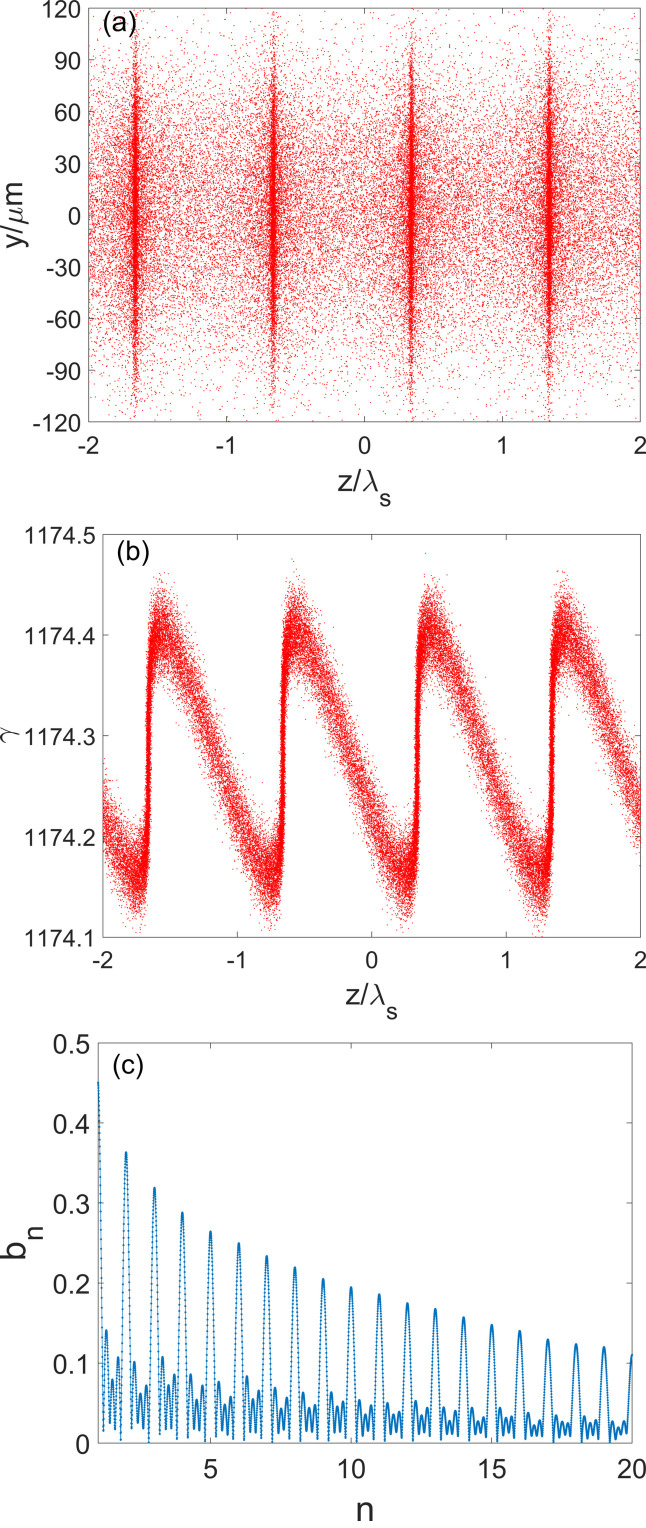
Simulation results of (*a*, *b*) the microbunching distribution and (*c*) bunching factors at various harmonics after the laser-beam interaction of ADM. λ_s_ is the seed laser wavelength.

To optimize the bunching factor at the 19th harmonic of the seed laser, the angles of all the bending magnets in the ADM section are set to be 1 mrad and the distance between dogleg bending magnets is 0.7 m. The longitudinal phase space of the electron beam and the bunching factors at different harmonics are shown in Fig. 5 ▸ at the exit of the ADM section, from which one can find that the energy spread after the ADM is about 40 keV, and the bunching factor at the 19th harmonic is approximately 12%.

**Figure 6:**
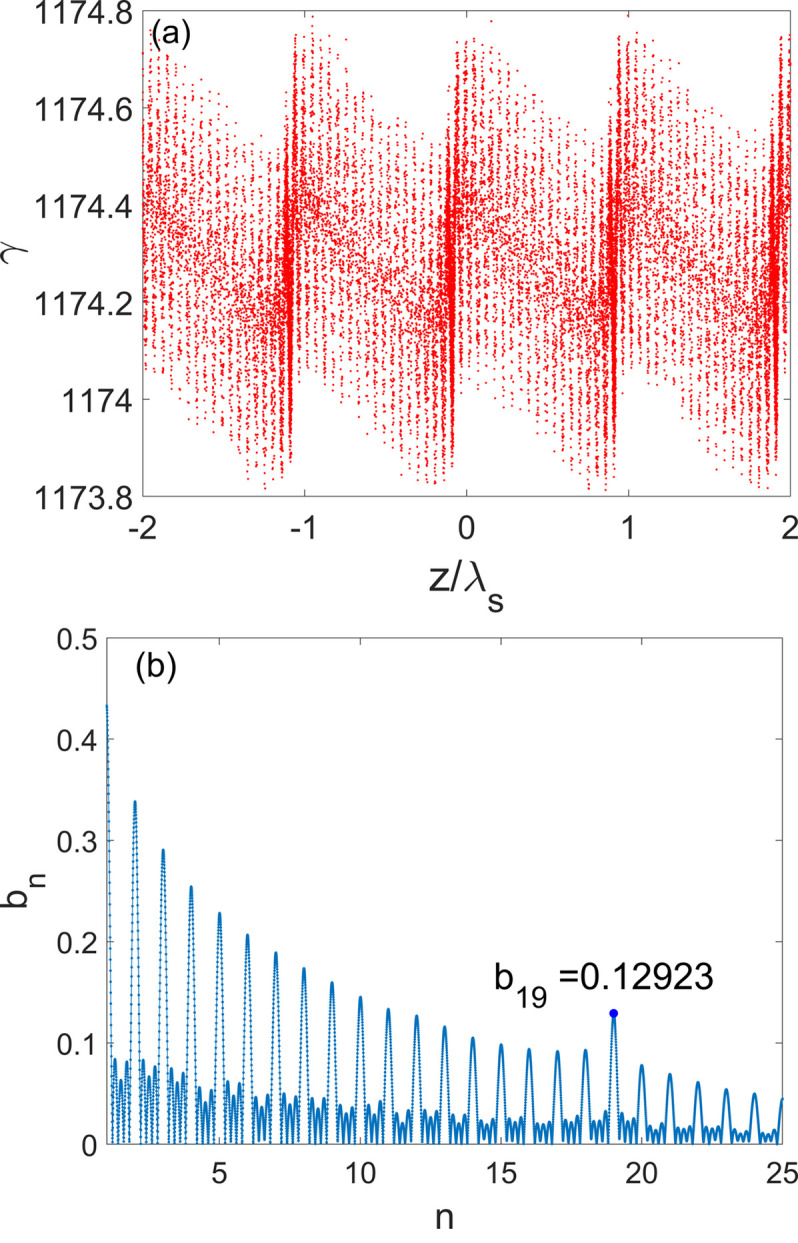
Simulation results of (*a*) the microbunching distribution and (*b*) bunching factors at various harmonics after the first radiation. λ_s_ is the seed laser wavelength.

**Figure 7:**
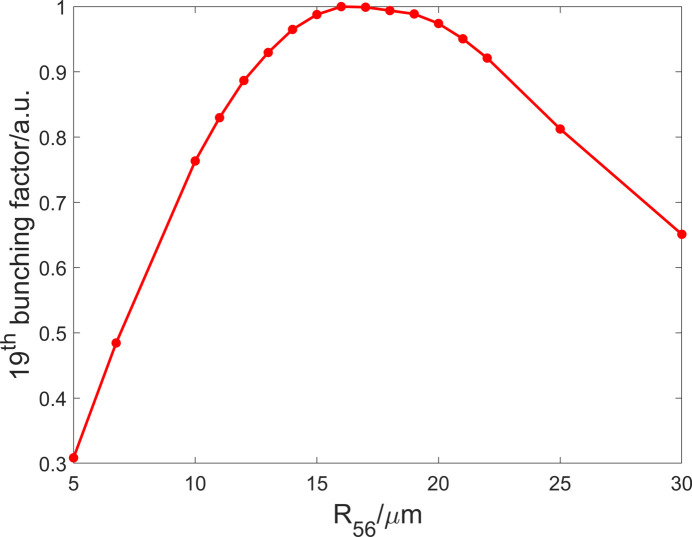
The 19th bunching factor of the beam after the first bending system with different *R*_56_ values (without nonlinear effects).

Subsequently, the electron beam is transported through the first 3.75 m-long radiator with a period length of 1.5 cm to generate fully coherent radiation at a wavelength of 13.5 nm, which is the 19th harmonic of the seed laser. The radiation will interact with the electron beam, leading to the continuous growth of the energy spread and radiation power. The energy modulation induced by the radiation is converted into the density modulation due to the dispersion of the undulator. Figure 6 ▸ illustrates the beam longitudinal phase space and the 19th bunching factor after the first radiator. As a result of the interaction between the radiation and the electron beam, the fine structures of microbunchings at 13.5 nm become apparent, the RMS energy spread increases to 92 keV, and the 19th bunching factor reaches approximately 13%. Initially, the bunching factor decreases due to *R*_56_ and then increases with the generation of fine structures. For short undulators, *R*_56_ can be calculated using *R*_56_ = −2*N*λ_r_ (Serkez *et al.*, 2017 ▸; Gensch *et al.*, 2011 ▸), where *N* is the undulator period number and λ_r_ is the resonant wavelength. However, for long undulators the physical mechanism becomes more complicated (Tsai *et al.*, 2023 ▸), rendering this simple equation inapplicable. Hence, it is imperative to conduct a simulation in which the electron beam travels the initial radiator and subsequently undergoes varying longitudinal dispersion strengths. Figure 7 ▸ illustrates the evolution of the 19th bunching factor after the first bending system with different values of *R*_56_ (without considering nonlinear effects and high-order effects) according to the simulation results. This figure can serve as a reference for determining the optimal *R*_56_ value for the downstream bending system, which falls within the range 13–22 µm. *R*_56_ of each bending system is chosen as 19.5 µm. One can optimize *R*_56_ of the second and subsequent bending system independently to further improve the radiation performance.

**Figure 8:**
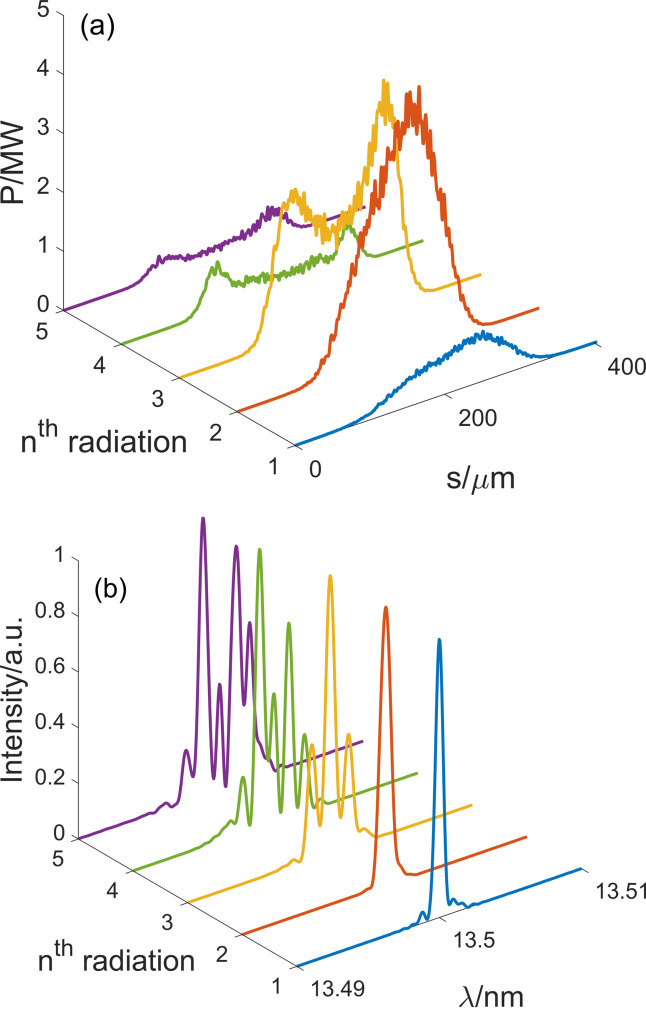
Radiation pulses (*a*) and spectra (*b*) at the exit of five radiators (3.75 m).

**Figure 9:**
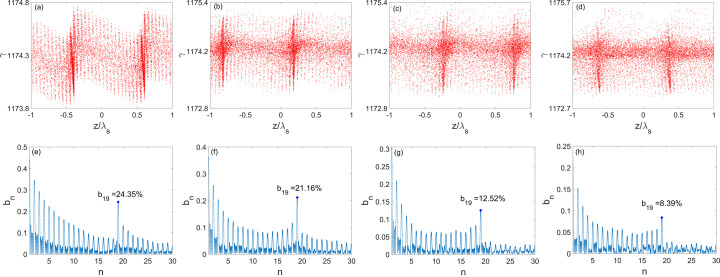
Simulation results of the longitudinal phase space (top) and bunching factors at various harmonics (bottom) at the exit of the first bending system [(*a*) and (*e*)], the second bending system [(*b*) and (*f*)], the third bending system [(*c*) and (*g*)] and the fourth bending system [(*d*) and (*h*)]. λ_s_ is the seed laser wavelength.

Then the electron beam passes through the designed bending system to achieve an 8 mrad bending angle in the horizontal and is sent through the second radiator to generate coherent radiation. The 8 mrad angle between the first and the second radiator improves the feasibility of the downstream beamline installation. Finally, the electron beam passes through the following bending systems and radiators to generate more radiation pulses at 13.5 nm. Figure 8 ▸ exhibits the radiation pulses and spectra at the exits of five radiators. The longitudinal phase space evolution of the electron beam and the bunching factors at various harmonics after passing through four bending systems are illustrated in Fig. 9 ▸.

As shown in Fig. 8 ▸(*a*), the peak power of the first radiation pulse is 0.84 MW and the average power is calculated as 540 W with 1.3 GHz repetition rate. The spectral bandwidth is approximately 0.0045% [full width at half-maximum (FWHM)], 1.09 times the Fourier transform limit.

After the first bending system, the bunching factor is enhanced to approximately 24% as a result of the opposite *R*_56_ value of the bending system. As can be seen in Fig. 9 ▸(*a*), the fine structure has been rotated to upright. It is remarkable that the bunching factor does not decrease in the presence of the CSR effect. The sufficiently large bunching factors indicate the suppression of the noise and the promotion of the coherent radiation in the radiators. In our study, the residual vertical dispersion at the entrance to the bending system does not affect the quality of beam bunching and radiation pulses.

**Figure 10:**
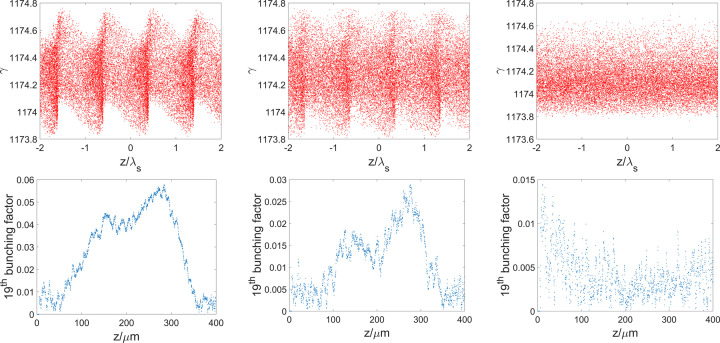
Simulation results of the longitudinal phase space (top) and 19th bunching factors (bottom) along the beam at the exit of the first-order four-bend achromat with *R*_56_ = −10 µm (left), the first-order triple bend achromat with *R*_56_ = 16 µm (middle) and the first-order isochronous achromat (right). λ_s_ is the seed laser wavelength.

The first-order achromats and isochronous lattices are also considered for the bending system to demonstrate possibility. A first-order four-bend achromat with *R*_56_ = −10 µm, a first-order triple-bend-achromat with *R*_56_ = 16 µm and a first-order isochronous achromat are designed. The total bending angles of these three lattices are also designed as 8 mrad. Figure 10 ▸ depicts the beam phase space (top) and the 19th bunching factor (bottom) along the beam after passing through the three lattices. It is evident that the debunchings are severe, with the 19th bunching factor dropping from 13% to 5.6%, 2% and 0% (noise level), respectively. The large *T*_5*ij*_ terms (at the 10^1^–10^3^ level) play critical roles in the microbunching smearing, which proves the necessity of eliminating these second-order terms.

The peak power of the second radiation pulse increases to 4.46 MW and the average power is calculated to be 2224 W. The spectral bandwidth is about 0.0063% (FWHM).

After passing through the second bending system, the bunching factor is well maintained. Figure 9 ▸(*b*) reveals obvious energy spread growth coming from the second radiator. The third radiation pulse has a peak power of 3.86 MW and a relatively longer pulse duration due to the beam splitting effect (Labat *et al.*, 2009 ▸), resulting in an average power of 6521 W with a repetition rate of 1.3 GHz. The spectral bandwidth is about 0.0053% (FWHM).

Figure 9 ▸(*c*) demonstrates the evident smearing of the microbunching and an increase in energy spread after the third bending system. Once the RMS value of the energy spread surpasses the adjacent energy bands, the fine structures will be erased because of the overlapping of the bands (Xiang *et al.*, 2009 ▸). This phenomenon is inevitable as the number of radiator segments increases. The second-order effects will also wash out the fine structures. Here, second-order terms apart from *T*_566_ are all eradicated and the major influence factor is second-order dispersion effects. Based on these reasons, the 19th bunching factor after the third bending system drops to 12% and the peak power of the fourth pulse is reduced to 0.84 MW. The average power is 748 W with a 1.3 GHz repetition rate. The spectral bandwidth is 0.019% (FWHM).

Figure 9 ▸(*d*) shows further impact of the second-order dispersion effect on the electron beam longitudinal distribution. The 19th bunching factor at the entrance to the fifth radiator is about 8.4%. Finally, the fifth pulse displays a peak power of 0.56 MW, and an average power of 425 W. The spectral bandwidth is about 0.027% (FWHM).

Generally, energy spread growth resulting from the radiation process, and second-order aberrations caused by the bending system are the predominant effects detrimental to the preservation of fine structures. However, despite these factors, the simulation results indicate that the final bunching factor remains relatively stable and comparable with its initial value. Furthermore, the radiation pulses are fully coherent with peak power levels of MW, manifesting the effectiveness of the proposed multiplexed emitting system.

## 4. Conclusions

In this paper, we propose and analyze a multiplexed emitting system that enables multi-beamline operation of an ERL-based fully coherent light source. Three-dimensional simulations show that the bunching factor of the electron beam first increases from 13% to 24% after one bend and then decreases to 8%, resulting in five radiation pulses with MW-level peak power at 13.5 nm wavelength. The sharp microbunching achieved after the bending system also indicates the possibility of increasing radiation pulse energy by two to three times. The technique studied in this paper requires high average power of the seed laser. However, the rapid development of laser technology in the last decade has brought it close to our needs (Gulliford *et al.*, 2013 ▸; Orii *et al.*, 2023 ▸). Additionally, new approaches to reduce the required power of the seed laser are being actively investigated. Moreover, this system can be optimized for different radiation wavelengths by adjusting the quadrupole and sextupole magnet strengths. This multi-point emitting system has the potential to significantly enhance the capabilities of ERL-based fully coherent light sources or free-electron laser facilities for supporting more beamlines simultaneously, which could stimulate new developments in scientific research and industrial applications.

## Figures and Tables

**Table 1:** Beam parameters at the injector exit and the ADM entrance

Parameter	Value	Units
Beam energy (injector)	15	MeV
Beam energy (ADM)	600	MeV
Slice RMS energy spread (ADM)	10	keV
Relative energy spread (ADM)	0.1	%
Normalized emittance (injector)	0.5	mm mrad
Normalized horizontal emittance (ADM)	5	mm mrad
Normalized vertical emittance (ADM)	0.05	mm mrad
Bunch charge	77	pC
Pulse duration (ADM, FWHM)	0.7	ps
Peak current (injector)	15	A
Peak current (ADM)	100	A

**Table 2:** Main parameters of the MBA

Parameter	Value	Units
Bend length	0.15	m
Bend angle	1	mrad
Quadrupole length	0.15	m
QF strength	3.785	m^−2^
QD strength	−3.785	m^−2^
Sextupole length	0.25	m
SF1 strength	1042	m^−3^
SF2 strength	1346	m^−3^
SD1 strength	−2968	m^−3^
SD2 strength	−682	m^−3^
*R*_56_	19.5	µm
*T*_566_	14.8	µm
